# Angiotensin II Evokes Angiogenic Signals within Skeletal Muscle through Co-ordinated Effects on Skeletal Myocytes and Endothelial Cells

**DOI:** 10.1371/journal.pone.0085537

**Published:** 2014-01-09

**Authors:** Jennifer L. Gorman, Sammy T. K. Liu, Dara Slopack, Khashayar Shariati, Adam Hasanee, Sara Olenich, I. Mark Olfert, Tara L. Haas

**Affiliations:** 1 School of Kinesiology and Health Science, Angiogenesis Research Group and Muscle Health Research Centre, York University, Toronto, Ontario, Canada; 2 West Virginia University School of Medicine, Center for Cardiovascular and Respiratory Sciences, Division of Exercise Physiology, Morgantown, West Virginia, United States of America; University of Sassari, Italy

## Abstract

Skeletal muscle overload induces the expression of angiogenic factors such as vascular endothelial growth factor (VEGF) and matrix metalloproteinase (MMP)-2, leading to new capillary growth. We found that the overload-induced increase in angiogenesis, as well as increases in VEGF, MMP-2 and MT1-MMP transcripts were abrogated in muscle VEGF KO mice, highlighting the critical role of myocyte-derived VEGF in controlling this process. The upstream mediators that contribute to overload-induced expression of VEGF have yet to be ascertained. We found that muscle overload increased angiotensinogen expression, a precursor of angiotensin (Ang) II, and that Ang II signaling played an important role in basal VEGF production in C2C12 cells. Furthermore, matrix-bound VEGF released from myoblasts induced the activation of endothelial cells, as evidenced by elevated endothelial cell phospho-p38 levels. We also found that exogenous Ang II elevates VEGF expression, as well as MMP-2 transcript levels in C2C12 myotubes. Interestingly, these responses also were observed in skeletal muscle endothelial cells in response to Ang II treatment, indicating that these cells also can respond directly to the stimulus. The involvement of Ang II in muscle overload-induced angiogenesis was assessed. We found that blockade of AT1R-dependent Ang II signaling using losartan did not attenuate capillary growth. Surprisingly, increased levels of VEGF protein were detected in overloaded muscle from losartan-treated rats. Similarly, we observed elevated VEGF production in cultured endothelial cells treated with losartan alone or in combination with Ang II. These studies conclusively establish the requirement for muscle derived VEGF in overload-induced angiogenesis and highlight a role for Ang II in basal VEGF production in skeletal muscle. However, while Ang II signaling is activated following overload and plays a role in muscle VEGF production, inhibition of this pathway is not sufficient to halt overload-induced angiogenesis, indicating that AT1-independent signals maintain VEGF production in losartan-treated muscle.

## Introduction

Communication between skeletal muscle fibers and the microcirculation is critical to ensure that the metabolic demands of the muscle are met. Angiogenesis, the process of blood vessel growth from pre-existing vessels, is induced in response to increased muscle activity, through the coordinated actions of growth factors and matrix degrading enzymes [1]–[3]. Matrix metalloproteinase (MMP)-2, which facilitates basement membrane degradation and capillary sprouting, is elevated in rodent muscle subjected to overload or electrical stimulation [2], [4] and in human muscle following exercise training [5]. Inhibition of MMP activity significantly attenuates the angiogenic response to chronic muscle activity [4]. Vascular endothelial growth factor (VEGF) plays a critical role in angiogenesis through promotion of endothelial cell proliferation and migration [6]–[9], and through regulating the production of MMPs [10], [11]. Reduction of VEGF signaling, either through competitive blockade of VEGFR activation (VEGF-TRAP) or by conditional deletion of myocyte VEGF, prevents capillary growth in response to muscle overload or exercise training, respectively [12], [13].

Recent studies have illustrated that cross-talk between myocytes and endothelial cells contribute to the regulation of angiogenesis. *In vitro* sprouting of microvessels and proliferation of cultured endothelial cells can be enhanced by co-culture with satellite cells [14], [15], or with conditioned media from satellite cells, a response which is attenuated when soluble VEGF receptors are added to the media [14]. VEGF not only promotes angiogenesis, but also is a regulator of myocyte differentiation and survival [16]. While diffusion of soluble VEGF originating from muscle cells may serve to activate endothelial cells, VEGF isoforms 164 and 188 are the predominant isoforms expressed in adult mouse skeletal muscle [16], and these isoforms have heparin binding domains that promote their interaction with the extracellular matrix (ECM) [17]. Matrix bound VEGF retains bioactivity, and has been shown to activate the p38 MAPK pathway in endothelial cells [17], [18]. Considering the close proximity of myocytes and capillary endothelial cells within skeletal muscle, matrix-associated VEGF may play an important role in crosstalk between the two cell types.

Transcriptional regulation of VEGF by HIF1α and PGC1α/ERRα is well established to occur under conditions of low oxygen tension, nutrient stress and in response to repeated bouts of exercise [19]–[21]. However, additional mechanisms may contribute to basal and activity-induced VEGF production within muscle [22]. Angiotensin II (Ang II) is implicated in both muscle remodeling and new capillary growth in response to muscle trauma, electrical stimulation, short-term training or overload [23]–[27]. Local production of Ang II within the muscle is likely to occur, given that myocytes produce all necessary components of the signaling pathway including angiotensinogen, the renin-like enzyme cathepsin D, angiotensin converting enzyme (ACE) and both angiotensin receptors (AT1R and AT2R) [28]–[30]. Ang II involvement in crosstalk between cultured myocytes and endothelial cells has been reported to occur by both direct and indirect signaling mechanisms. Ang II stimulation of endothelial cells induces network formation, which can be blocked by the AT1R inhibitor losartan [25]. A similar response is evoked by exposing endothelial cells to conditioned media from C2C12 myocytes, and this is attenuated by pre-treatment of C2C12 cells with the ACE inhibitor captopril [25], suggesting that myocytes release Ang II, which then exerts angiogenic effects directly on endothelial cells. Alternatively, Ang II has been reported to induce VEGF production in retinal pericytes and smooth muscle cells [31], [32] and has been correlated with VEGF release from cardiac myocytes [33]. Thus, Ang II may promote angiogenesis indirectly by acting first on skeletal myocytes to induce VEGF production, which in turn would activate the angiogenic cascade within neighbouring endothelial cells.

The importance of muscle-derived VEGF to muscle overload-induced angiogenesis and the contribution of the Ang II signaling to this process has yet to be fully ascertained. We hypothesized that muscle-derived VEGF plays a critical role in the angiogenic response to muscle overload and that the Ang II signaling pathway is an important upstream mediator of myocyte-endothelial cell communication and overload-induced capillary growth.

## Methods

### Ethical Approval

Animal studies were carried out with approval from the York University Committee on Animal Care and performed in accordance with the animal care procedures at York University and the American Physiological Society’s guiding principles in the care and use of animals.

### Materials

Chemicals, including Ang II, were purchased from Sigma-Aldrich Canada (Oakville, Ontario) and losartan was obtained from LKT Laboratories (St. Paul, MN), while the VEGFR2 inhibitor SU4312 was purchased from Santa Cruz Biotechnologies (Santa Cruz, CA) and the AT2R agonist (CGP42112) and antagonist (PD123319) were purchased from Cedarlane Laboratories (Mississauga, ON).

### Animal Studies

#### Mice

Muscle VEGF KO mice (*VEGFLoxP* mice on a C57Bl/6 background crossbred with *Myo-Cre* mice expressing cre recombinase under the control of the muscle creatine kinase promoter) [12], [34] and littermate controls (*VEGFLoxP*) were bred and genotyped at the West Virginia animal facility and then transferred to the animal facility at York University. Mean age of the mice at the time of experiment was 66+/−3 days (*VEGFLoxP*) and 70+/−3 days (*Myo-Cre:VEGFLoxP*). Overload of the extensor digitorum longus (EDL) muscle was induced through unilateral extirpation of the tibialis anterior (TA) muscle [3], while animals were under isoflurane anesthesia. Sham surgeries consisted of TA exposure without its removal, followed by suturing of the overlying skin. WT (*VEGFLoxP*) and KO (*Myo-Cre:VEGFLoxP*) animals were divided into 4 groups: 7 day Sham (n = 4 for WT and KO), 7 day OL (n = 3 for WT and n = 4 for KO), 14 day Sham (n = 10 for WT and n = 4 for KO) and 14 day OL (n = 7 for WT and n = 5 for KO). After 7 or 14 days, animals were anaesthetized with isoflurane and the EDL muscle was removed for further analysis. C57BL/6 mice were used to assess angiotensinogen levels. Sham and overload surgeries were performed as described above. Animals were divided into 2 groups sham (n = 3) and overload (n = 6). After 5 days, animals were anesthetized with isoflurane and the EDL muscle was removed for further analysis.

#### Rats

Male Sprague-Dawley rats (approximately 375 g at sacrifice; Charles River Laboratories, Quebec, Canada) were used to test the effect of AT1R blockade on overload-induced angiogenesis. Sham and overload surgeries were performed as described for mice. Rats were divided into 4 groups; sham + vehicle (n = 4), overload + vehicle (n = 4), sham + Losartan (n = 4) and overload + Losartan (n = 4). Losartan (20 mg/kg/day) was delivered in the drinking water [35]–[38]. Treatment began 4 days in advance of the overload surgery, and continued throughout the duration of the experiment. After 14 days, all rats were anaesthetized (i.p. injection of 80 mg/kg ketamine and 10 mg/kg xylazine) and the EDL muscle was removed for further analysis.

### Capillary Number

Capillary to muscle fiber ratio was assessed as an indicator of angiogenesis. 10 µm cross-sections of frozen mouse or rat EDL muscle were fixed in cold acetone and stained with FITC-conjugated Griffonia simplicifolia I isolectin (1∶100; Vector Labs) for 30 minutes. Sections were viewed on an Olympus microscope (x20 objective). Capillary and muscle fiber counts were averaged from 10 independent fields of view per rat and 5 independent fields of view per mouse.

### Cell Culture

Primary rat microvascular endothelial cells were isolated from the EDL and TA muscles, as previously described [39] and the C2C12 cells were a kind gift from Dr. David Hood, York University. Both cell lines were cultured under conditions of 37°C and 6% CO_2_ on 1.5% gelatin coated flasks with Dulbecco’s Modified Eagle Medium (DMEM) supplemented with 10% fetal bovine serum, 1 mM sodium pyruvate, 200 mM L-glutamine, 50 units of penicillin and 0.5 mg/ml streptomycin. In some experiments, C2C12 myoblasts were differentiated to myotubes in DMEM supplemented with 2% horse serum for 5–8 days. Endothelial cells or C2C12 myotubes were stimulated with Ang II (0.1 µM or 1 µM) or treated with the AT1R inhibitor losartan (0.1 and 1 µM) for the indicated duration. In some experiments, endothelial cells were treated with the AT2R agonist CGP42112 (100 nM) or the AT2R antagonist PD123319 (1 µM) for 24 hours.

### Preparation of C2C12 Cell-Free Matrix

Myoblasts were cultured to confluency on type I collagen-coated 60 mm dishes. Cells were removed from the type I collagen coated culture dishes first by incubation with 2 M urea in DMEM (10 min at 37°C), followed by PBS washes and a 3 minute incubation with extraction buffer containing 0.5% Triton X-100 and 20 mM ammonium hydroxide [40]. Matrix proteins were washed extensively with 1X PBS prior to utilization for Western blotting or in matrix-endothelial cell experiments.

### Matrix Experiments

C2C12 myoblasts were plated on type I collagen and cultured to confluence (48 hours), then incubated overnight in Optimem or Optimem containing the AT1R inhibitor losartan (0.1 µM). Endothelial cells were plated on a type I collagen-coated coverslip and incubated overnight in either media alone or media containing the VEGFR inhibitor SU4312 (4 µM). Following removal of C2C12 cells to generate a cell-free matrix (as described earlier), endothelial cell coverslips were overlaid on the cell cleared matrix for 5 minutes (cell side in contact with matrix), followed by protein lysis.

### Protein Extraction

#### Muscle

Frozen tissue was ground to powder and protein was isolated through brief homogenization in buffer containing 120 mM Tris HCl, 5% glycerol and protease inhibitor cocktail (Roche). After homogenization, Triton X-100 (Sigma) was added to a final concentration of 0.1%, as described previously [4].

#### Endothelial and myotube cell lysates

Cells were lysed in 120 mM Tris HCl, 0.1% Triton X-100 and 5% glycerol or in RIPA buffer supplemented with 0.1% EDTA-free protease inhibitor cocktail (P-8340, Sigma), 2 mM sodium orthovanadate, 1 mM sodium fluoride and 1 mM PMSF. The protein concentration of all lysates was determined using the bicinchonic acid assay (Pierce).

*Matrix proteins* – After preparation of cell-free matrix, matrix bound proteins were lysed directly in 1X Laemmli sample buffer (38 mM Tris, 65 mM DTT, 4% SDS, 5% glycerol).

### Western Blotting

Protein samples were prepared under denaturing conditions and separated on a polyacrylamide gel. Proteins were transferred to a PVDF membrane (Millipore) and incubated in either 5% milk or 5% BSA. Membranes were probed for angiotensinogen (1∶1000, Ab108334 Abcam, Toronto, ON), AT1R (1∶200, sc-1173 Santa Cruz), AT2R (1∶2000, sc-7420 Santa Cruz), VEGF (1∶200, sc-152 Santa Cruz or 1∶200, sc-57496 Santa Cruz), phospho Ser473-Akt (1∶1000, #4051 Cell Signaling; NEB Canada, Whitby, ON), total Akt (1∶1000, #9272 Cell Signaling), p-p42/p44 (1∶1000, #9101 Cell Signaling), p-p38 (1∶1000, #9211 Cell Signaling), ß-actin (1∶1000, #4967 Cell Signaling) or α/ß tubulin (1∶1000, #2148 Cell Signaling) overnight at 4°C. Membranes were incubated with anti-rabbit (GE Biosciences; Baie d’Urfe, Quebec, or Pierce; Fisher Scientific, Whitby ON) or anti-mouse (Dako; Cedarlane, Burlington, Ontario) HRP secondary antibodies. Bound antibodies were detected using Super West Pico (Pierce; Fisher Scientific, Whitby ON) or Immobilon (Millipore; Fisher Scientific, Whitby ON) ECL and were visualized using a digital imaging system (Kodak MM4000Pro) or imaged on autoradiography film (Hyperfilm, GE Biosciences). Bands were quantified using Fluorchem (AlphaInnotech) or Carestream (Kodak) software.

### RNA Extraction and qPCR

EDL muscle was lysed using a Retsch MM400 tissue lyser (GmbH, Haan, Germany) and RNA was isolated using the RNeasy Fibrous Tissue Mini Kit (Qiagen), using manufacturer’s instructions. Cultured cells were lysed using cells-to-cDNA II reagent (Ambion) according to manufacturer’s instructions, followed by DNase treatment. RNA was reverse transcribed using M-MuLV reverse transcriptase (NEB Canada). cDNA samples were analyzed by real time PCR using Universal Fast-PCR mix or Universal PCR mix (Applied Biosystems) and Taqman probe/primer sets (Applied Biosystems - Rat: GAPDH - Rn99999916_S1, VEGF - Rn00582935_m1 and MMP-2 - Rn02532334_s1. Mouse: MT1-MMP - Mm00485054_m1, GAPDH - Mm03302249_g1, VEGF - Mm00437306_m1, MMP-2 Mm00439498_m1, Angiotensinogen - Mm00599662_m1, Angiotensin type 1a receptor -Mm01957722_s1, HPRT1 -Mm00446968_m1 ). Real time PCR was performed using an ABI Prism 7700 Sequence Detector (Perkin Elmer) and results were analyzed using associated software. The threshold cycle (Ct) was determined and normalization was carried out using the comparative Ct method, with GAPDH as the housekeeping gene, as previously described [41]. The average Ct for all samples was used as the comparator, which was set to 1.

### RT-PCR Screen of VEGF Isoforms

Endothelial cells and C2C12 myotubes were lysed using cells to cDNA reagent (Ambion) without DNase treatment. PCR conditions were 98°C for 30 seconds followed by 30 cycles of 98°C for 10 seconds, 68°C for 30 seconds and 72°C for 60 seconds followed by incubation at 72°C for 5 minutes. Primers were designed using the rat VEGF sequence (Accession # NM_031836.2) with the forward primer located in exon 4, 5′-GAGATGAGCTTCCTACAGGAC-3′ and the reverse primer located in exon 9, 5′-GGTGACATGGTTAATCGGTCTTTC-3′. The expected product sizes were 171 bp for VEGF_120_, 300 bp for VEGF_164_, and 372 bp for VEGF_188_.

### Statistics

Data are presented as mean ± SEM. Comparisons between groups were performed using Student’s t-test, one or two way ANOVA followed by Tukey’s or Bonferroni’s multiple comparison tests with GraphPad Prism 4. Statistical significance was set at p<0.05.

## Results

### Loss of Myocyte VEGF Prevents Overload-induced Angiogenesis

Overload of the extensor digitorum longus (EDL) results in significantly enhanced production of pro-angiogenic factors after 4–7 days, and detectable increases in capillary number after 14 days [1], [2], [41], [42]. Previously, skeletal muscle deletion of VEGF was shown to significantly reduce basal capillary number in skeletal muscle and to impair the exercise-induced angiogenesis response [12], [34]. To assess the importance of muscle VEGF to overload-induced angiogenesis, we examined mRNA levels of pro-angiogenic factors VEGF, MMP-2 and MT1-MMP after 7, and capillary growth after 14, days of EDL overload in wildtype or muscle VEGF-deficient mice.

**Figure 1:**
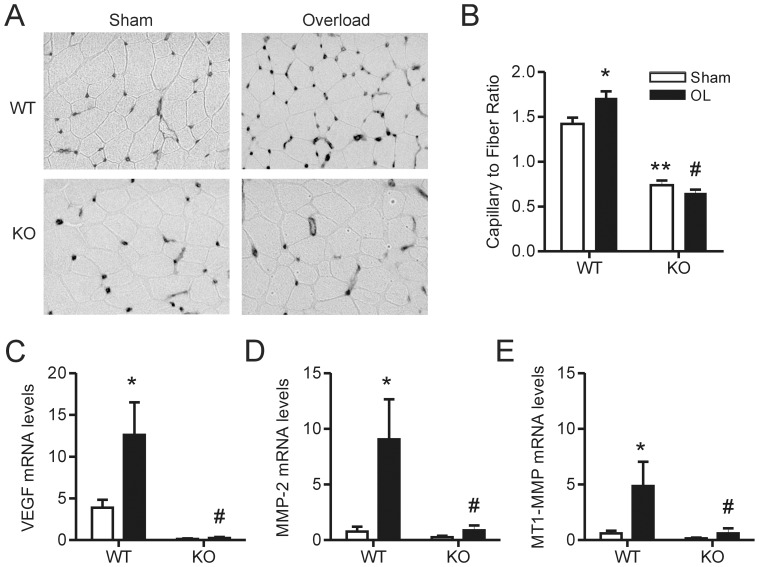
Muscle overload-induced angiogenesis is attenuated in muscle VEGF KO animals. Representative inverted grey-scale images of iso-lectin staining (A) were used to calculate the capillary to muscle fiber ratio (n = 10 for WT Sham, n = 4 for KO Sham, n = 7 for WT OL and n = 5 for KO OL) (B). Muscle subjected to overload for 7 days was lysed for qPCR analysis of VEGF (C), MMP-2 (D) and MT1-MMP (E) transcript levels (n = 4 for WT Sham, KO Sham and KO OL and n = 3 for WT OL). Values are presented as mean ± SEM. There was a significant main effect of overload on the capillary to fiber ratio as well as VEGF, MMP-2 and MT1-MMP transcript levels, as assessed by two way ANOVA (*). Bonferroni’s multiple comparison posthoc tests revealed a significant difference between the capillary to fiber ratio of KO sham animals compared WT sham animals (**) and a significant difference in the capillary to fiber ratio and VEGF, MMP-2 and MT1-MMP transcript levels in KO overload animals compared to WT overload animals (#). WT – wildtype, KO – knockout and OL – overload.

14 days of EDL muscle overload significantly increased the capillary-to-muscle fiber ratio in wild-type mice (Figure 1A, B) and this response was abolished in muscle VEGF knockout mice (Figure 1A, B). We observed the expected increase in VEGF mRNA [42] following overload in wild-type animals, while muscle VEGF-deficient mice exhibited no VEGF response to the overload stimulus (Figure 1C). Consistent with previous observations in rats [2], [41], muscle overload significantly increased both MMP-2 and MT1-MMP mRNA expression in wild-type animals (Figure 1D, E). Previously, we reported that VEGF induces the expression of these proteases in endothelial cells [10], [11]. In agreement with those findings, we observed that the MMP-2 and MT1-MMP mRNA responses to muscle overload were attenuated significantly in muscle VEGF-deficient mice (Figure 1D, E).

### Effect of Skeletal Muscle Overload on Angiotensinogen Expression

**Figure 2:**
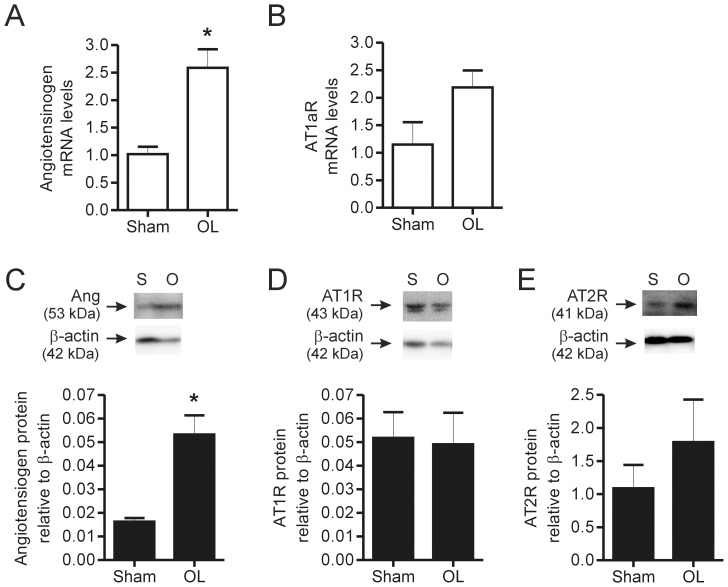
Effect of skeletal muscle overload on angiotensinogen and AT receptor expression in skeletal muscle. Muscle subjected to overload for 5 days was lysed for qPCR analysis of angiotensinogen (A) and AT1aR (B) transcript levels. Protein levels of Angiotensinogen (C), AT1R (D) and AT2R (E) were assessed by Western Blotting. In each graph, values are presented as mean ± SEM (n = 3 for Sham and n = 6 for Overload). Student’s t-test (*) revealed a significant difference between angiotensinogen transcript and protein levels in overload compared to sham animals. OL – overload, Ang – angiotensinogen, AT1R – Ang II type 1 receptor and AT2R – Ang II type II receptor.

Skeletal muscle is reported to produce components of the renin-angiotensin system, which can lead to the local production of Ang II [28], [29]. Increased production of angiotensinogen has been observed in response to muscle stretch in both proliferating and differentiated C2C12 cells [28]. As those experiments were carried out in culture, we assessed whether components of the renin-angiotensin system were modified in response to skeletal muscle overload in the mouse. We examined mRNA levels of angiotensinogen and the AT1a receptor in EDL that was overloaded for 5 days. We observed a significant increase in angiotensinogen mRNA levels in whole muscle homogenates (Figure 2A), indicating that it is responsive to muscle overload. However, AT1aR levels (Figure 2B) were unchanged in response to muscle overload. Similar to the mRNA levels, overloaded muscle expressed a higher level of angiotensinogen protein in comparison to sham muscle (Figure 2C). Conversely, protein levels of both AT1R and AT2R were detectable but not altered by muscle overload (Figure 2D,E).

### VEGF-matrix Interactions Contribute to Endothelial Cell and Muscle Signaling

**Figure 3:**
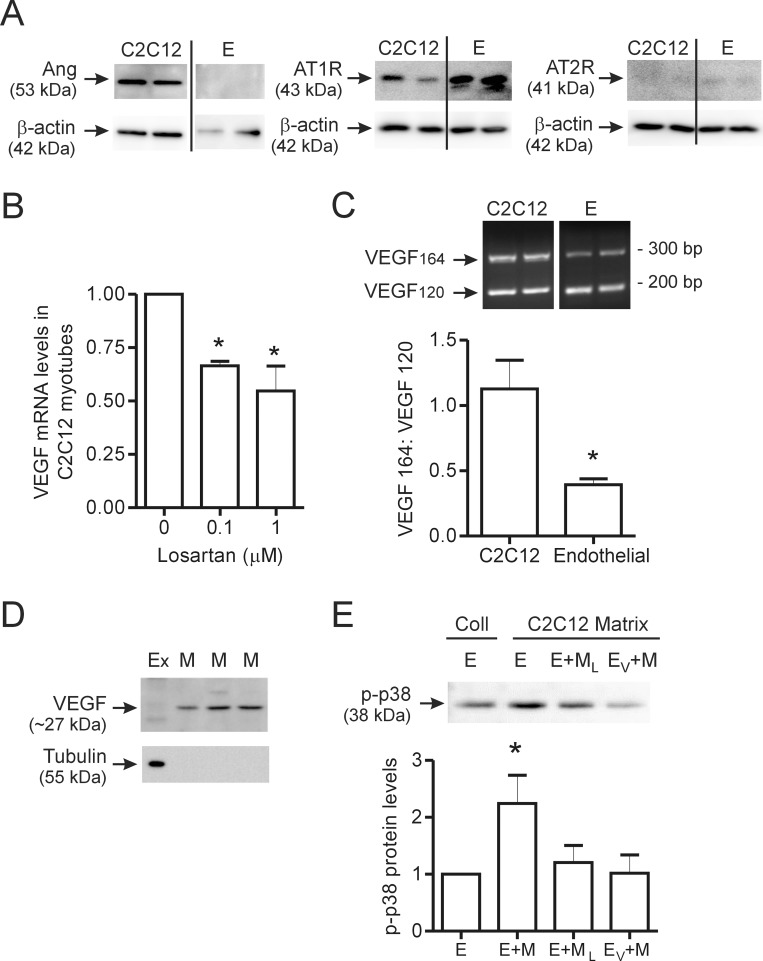
Contribution of endogenous Ang II to muscle VEGF production and endothelial cell-muscle cell crosstalk. C2C12 myotubes and confluent cultures of microvascular endothelial cells were lysed, and endogenous basal levels of angiotensinogen, AT1R and AT2R were assessed by Western blot analysis (A). In all blots, two independent cultures are shown for each cell type. C2C12 myotubes (n = 3 per condition) were treated overnight with the AT1R inhibitor Losartan (0.1 µM or 1 µM) and VEGF transcript levels were assessed by qPCR (B). Representative image and the quantification of RT-PCR analysis of VEGFA isoform expression (VEGF_120_ ∼170 bp; VEGF_164_ ∼300 bp and VEGF_180_ = not detected) in C2C12 myotubes and skeletal muscle endothelial cells (C). Two independent samples are shown for both C2C12 and endothelial cells (n = 6 for C2C12 and n = 13 for endothelial cells). Lysates of matrix bound proteins were assessed for VEGF and tubulin protein expression by Western blotting (D). Cell extract (Ex) was used as a comparator with extracts of matrix (M) alone. Three independent samples of matrix-associated protein extracts are shown. Tubulin was detectable within cell extract, but not in matrix-derived extracts. In (E), endothelial cells alone or previously treated with an inhibitor of the VEGFR2, were incubated on matrix which previously contained C2C12 myoblasts with or without losartan treatment. Representative image and quantification of phosphorylated p38 levels (n = 6 for E and E+M, n = 5 for E+M_L_ and n = 4 for E_V_+M). Values are presented as mean ± SEM. One way ANOVA followed by Tukey’s multiple comparison test and student’s t-test were used to assess statistical significance which was set as p<0.05. In panel B, *denotes p<0.05 vs. untreated cells. In panel C, *denotes p<0.05 vs. C2C12 cells and in panel E, *denotes p<0.05 vs. endothelial cells. E – endothelial cell, Ex - cell extract, Coll- type I collagen matrix, M - matrix lysate, E+M – endothelial cells + matrix from untreated C2C12, E+M_L_ – endothelial cells + matrix from losartan-treated C2C12 cells and E_V_+M – endothelial cells pretreated with a VEGFR2 inhibitor + matrix from untreated C2C12.

Next, we examined whether endogenous Ang II signaling plays a role in the basal production of VEGF in C2C12 myotubes. Angiotensinogen was produced by C2C12 cells under resting conditions, but not detectable in cultured endothelial cells (Figure 3A). C2C12 cells also predominantly expressed the AT1R, while AT2R expression was minimally detectable. As Ang II modulation of VEGF expression in cardiac endothelial cells occurred via the AT1 receptor [43], we treated cells overnight with the AT1 receptor inhibitor losartan. Losartan treatment (0.1 and 1.0 µM) significantly reduced basal VEGF mRNA expression in C2C12 myotubes (Figure 3B). The predominant VEGF isoforms that are reported to be produced within skeletal muscle [16] have high affinity for heparin sulphate moieties within matrix proteins, and as such, are likely to be immobilized by matrix rather than freely diffusible within the interstitial fluid. We assessed the VEGF isoforms produced by C2C12 myotubes and compared them to the isoforms produced by cultured endothelial cells, using RT-PCR. VEGF isoforms 120 and 164 were detectable within both cultured C2C12 myotubes and skeletal muscle endothelial cells (Figure 3C). However, the relative levels of VEGF164 compared to VEGF120 were significantly higher in myotubes compared to endothelial cells (Figure 3C). VEGF188 was undetectable in endothelial cells, and was observed infrequently in C2C12 myocytes.

To detect the matrix-bound fraction of VEGF, myoblasts were cultured on type I collagen overnight, then cells were removed to enable analysis of the cell-free matrix-associated proteins. Western blotting of matrix deposited by unstimulated C2C12 cells revealed minimal VEGF (21 kDa) within the cell lysate, and substantial levels of VEGF (27 kDa) associated with the matrix (Figure 3D). Lack of tubulin immune-reactivity indicated that cellular components did not contribute to the VEGF signal observed in matrix lysates (Figure 3D). These results demonstrate that, under basal conditions, skeletal muscle myocytes actively produce VEGF that is able to bind and accumulate within the underlying matrix. Finally, we assessed the bioactivity of the matrix-associated VEGF and whether Ang II signaling played a role in the secretion of matrix binding variants of VEGF. Activation of the VEGFR2 in endothelial cells is known to cause the phosphorylation of p38 MAPK [18], [44]. Thus, we analyzed endothelial cell phosphorylation of p38 MAPK (p-p38) as an indicator of VEGFR2 activation. Direct contact of endothelial cells with the cell-free C2C12 matrix resulted in an increase in p38 phosphorylation, as compared to endothelial cells incubated on type I collagen matrix that had not been exposed previously to C2C12 cells (Figure 3E). These results indicate that matrix bound VEGF deposited by C2C12 cells is able to activate endothelial cells. Incubation of endothelial cells on matrix derived from C2C12 cells that had been pre-treated with losartan resulted in decreased p-p38 levels as did pre-treatment of the endothelial cells with an inhibitor of the VEGFR2 (Figure 3E). Thus, deposition of matrix-associated VEGF by C2C12 cells is sensitive to AT1R inhibition and the induction of p38 phosphorylation in endothelial cells occurs via the VEGFR2.

### Effect of Ang II on VEGF Expression in Skeletal Myocytes and in Microvascular Endothelial Cells

**Figure 4:**
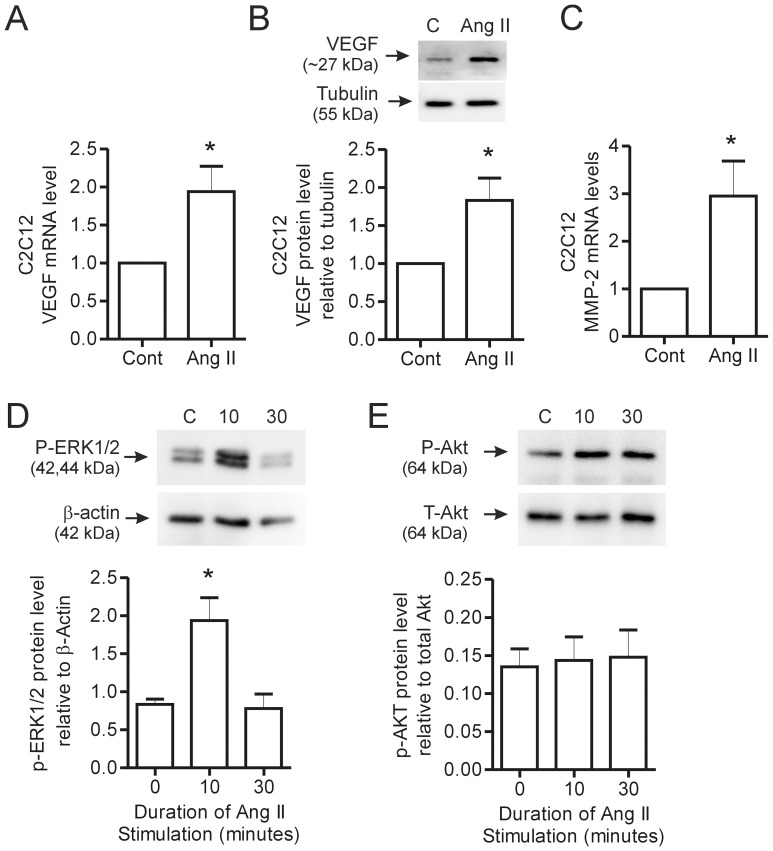
Effect of Ang II stimulation on VEGF and MMP-2 expression in C2C12 myotubes. C2C12 myoblasts were differentiated in 2% serum media for 5–8 days until myotubes had formed. Following myotube formation, cells were switched to Opti-mem reduced serum media and stimulated with Ang II. VEGF mRNA levels were determined by qPCR in response to Ang II (0.1 µM) stimulation for 2 hours (n = 7 per condition) (A). Representative blot and quantification of VEGF protein expression after overnight stimulation with Ang II (0.1 µM) and normalized to tubulin (n = 7 per condition) (B). MMP-2 mRNA levels were assessed in response to 2 hour Ang II (0.1 µM) treatment by qPCR (n = 6 per condition) (C). C2C12 myoblasts were stimuated for either 10 or 30 minutes with 1 µM Ang II and then lysed for protein analysis. P-ERK1/2 (n = 3 per condition) (D) and P-Akt (n = 5 per condition) (E) levels were assessed by Western blotting with activated levels normalized to ß-actin and total Akt levels respectively. Values are presented as mean ± SEM. Student’s t-test and one way ANOVA followed by Bonferroni’s multiple comparison test were used to assess statistical significance which was set as p<0.05. In panels A–D, *denotes p<0.05 vs. untreated cells. C – Control, 10–10 minutes and 30–30 minutes.

The above results suggest that endogenous Ang II contributes to VEGF production by skeletal myocytes. To extend these findings, we treated C2C12 myotubes with exogenous Ang II. Ang II treatment (0.1 µM) stimulated a significant increase in VEGF mRNA (Figure 4A) and protein expression (Figure 4B), as well as an increase in MMP-2 transcript levels (Figure 4C) in C2C12 myotubes. Ang II (1 µM) induced the phosphorylation of ERK1/2 after 10 minutes, with levels returning to those observed in control cells by 30 minutes (Figure 4D). Akt phosphorylation was not affected by Ang II treatment (Figure 4E).

**Figure 5:**
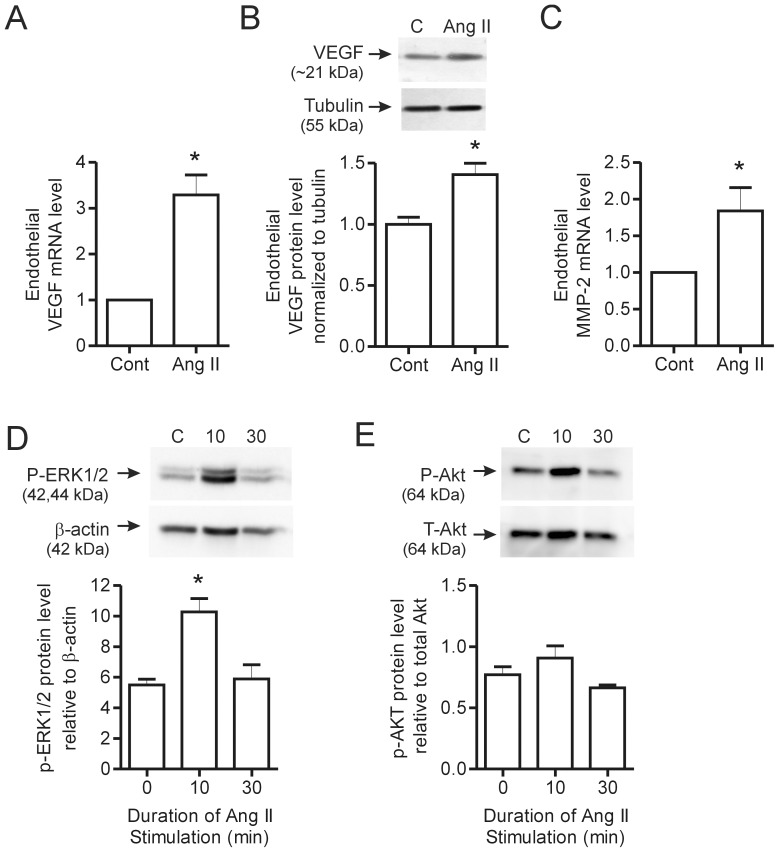
Effect of Ang II stimulation on VEGF and MMP-2 expression in skeletal muscle endothelial cells. Skeletal muscle endothelial cells were stimulated for 2 hours with Ang II (0.1 µM) and lysed for qPCR analysis of VEGF (A) and MMP-2 (C) transcript levels (n = 6 per condition). In (B), endothelial cells were treated overnight with Ang II (0.1 µM) and Western blotting was performed to assess VEGF protein levels, which were normalized to tubulin (n = 3 per condition). Endothelial cells were stimulated for either 10 or 30 minutes with 1 µM Ang II and then lysed for protein analysis. P-ERK1/2 (D) and P-Akt (E) levels were assessed by Western blotting and normalized to ß-actin and total Akt levels respectively (n = 3 per condition). Values are presented as mean ± SEM. One way ANOVA followed by Tukey’s multiple comparison test and student’s t-test were used to assess statistical significance (p<0.05). In panels A–D, *denotes p<0.05 vs. untreated cells. C – Control, 10–10 minutes and 30–30 minutes.

Given that endothelial cells also expressed AT1R and AT2R (Figure 3A), we also assessed the direct effects of exogenous Ang II on the same factors within cultured primary microvascular endothelial cells. Stimulation of microvascular endothelial cells with Ang II (0.1 µM) resulted in a significant increase in VEGF mRNA expression after 2 hours (Figure 5A), with protein levels elevated following overnight Ang II treatment (Figure 5B). MMP-2 mRNA also was increased significantly by Ang II stimulation (Figure 5C). Similar to the response observed in C2C12 cells, Ang II (1 µM) induced the transient phosphorylation of ERK1/2 (Figure 5D) but did not alter Akt phosphorylation (Figure 5E) in endothelial cells, suggesting the involvement of ERK1/2 in mediating Ang II signaling.

### Role of Ang II in the Angiogenic Response to Muscle Overload

**Figure 6:**
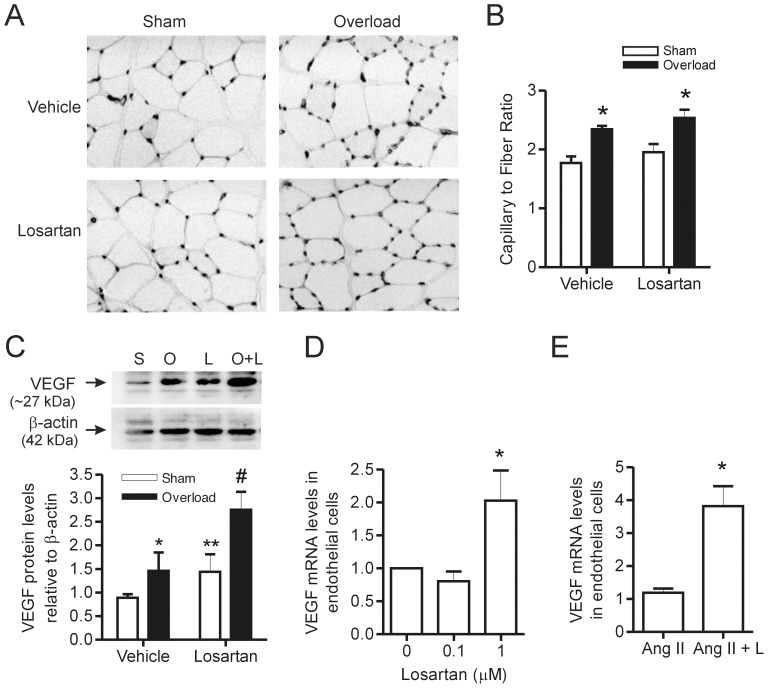
Overload-induced angiogenesis is not altered by losartan treatment. Representative inverted grey scale images of iso-lectin staining (A) were used to calculate capillary to muscle fiber ratio (n = 4 per condition) (B). In (C), VEGF protein levels were assessed by Western blotting with expression normalized to ß-actin (n = 3 for sham + vehicle, n = 4 for overload + vehicle, overload + losartan, and for sham + losartan). Skeletal muscle endothelial cells (n = 4 per condition) (D) were treated overnight with the AT1R inhibitor Losartan (0.1 or 1 µM) and in (E) cells were treated with Ang II (0.1 µM) or Ang II and losartan (1 µM) overnight. Cells were lysed for qPCR analysis of VEGF mRNA levels. Values are presented as mean ± SEM. There was a significant main effect of overload on capillary to muscle fiber ratio (*), as assessed by two way ANOVA. For VEGF protein levels, two way ANOVA revealed a significant main effect of overload (*) and the losartan treatment (**) as well as a significant difference between overload and overload + losartan conditions and the losartan and overload + losartan conditions (#) by Bonferroni’s multiple comparison test. In (D), *denotes p<0.05 vs. untreated cells and in E, *denotes p<0.05 vs. Ang II treatment alone. S-sham, O-overload and L-losartan.

Based on our observations that VEGF released from skeletal muscle is critical to overload-induced angiogenesis (Figure 1A, B), the Ang II signaling pathway is activated in muscle subjected to overload (Figure 2A) and that Ang II contributes to basal VEGF production in myocytes (Figure 3A), we hypothesized that AT1R inhibition would reduce muscle VEGF levels and impair the angiogenic response to muscle overload. Animals were treated daily with the AT1R inhibitor losartan (provided in the drinking water), beginning 4 days prior to the induction of overload. Losartan treatment did not attenuate the overload-induced increase in capillary to muscle fiber ratio (Figure 6A, B). In line with a previous study in mice [45], VEGF protein expression in whole muscle homogenates was elevated following 7 days of muscle overload (Figure 6C). However, there was a significant effect of the losartan treatment alone on basal VEGF expression and the combination of overload and losartan further increased VEGF expression levels compared with losartan treatment alone, indicating a possible additive effect of the two treatments.

To elucidate the apparent discrepancy between *in vitro* and *in vivo* findings, we examined the effects of AT1R signaling inhibition on cultured microvascular endothelial cells. Treatment of endothelial cells with 0.1 µM losartan had no effect on basal VEGF expression, while 1.0 µM losartan significantly elevated endothelial cell VEGF expression (Figure 6D). The increased VEGF expression in endothelial cells treated with losartan was unexpected, so we assessed the combined effect of Ang II and losartan treatment in endothelial cells. VEGF mRNA levels were increased significantly in endothelial cells treated with both Ang II and losartan compared to endothelial cells treated with Ang II alone (Figure 6E). These results reveal that inhibition of AT1R signaling contributed a stimulatory, rather than inhibitory, effect on Ang II-induced VEGF mRNA levels in endothelial cells. This influence did not appear attributable to the actions of the AT2R, as treatment of endothelial cells with the AT2R agonist CGP 42112 failed to induce an influence on VEGF mRNA levels (1.0±0 vs. 0.93±0.1; p = 0.5, n = 6), and treatment with an AT2R antagonist (PD123319) did not prevent the losartan-induced effect on VEGF mRNA (2.9±0.4 vs. 3.1±0.7; p = .8, n = 6).

## Discussion

We provided conclusive evidence that muscle-derived VEGF is an absolute requirement for overload-induced skeletal muscle angiogenesis. We also established that angiotensinogen mRNA is elevated following muscle overload and that endogenous Ang II regulates basal production of VEGF in cultured myocytes. Despite our finding that exogenous treatment of both myotubes and endothelial cells with Ang II increased levels of both VEGF and MMP-2 mRNA expression, we found that inhibition of the AT1R *in vivo* does not impair the overload-induced increase in VEGF expression and new capillary growth.

Studies utilizing mice with deficiency of VEGF in skeletal muscle have provided evidence that muscle-derived VEGF is a critical regulator of basal muscle capillarity as well as exercise-induced angiogenesis [12], [34]. Consistent with these reports, we did not detect capillary growth within overloaded muscle of mice with myocyte-specific VEGF deficiency. As expected, VEGF mRNA levels were substantially reduced within muscle extracts of VEGF knockout mice. Multiple cell types within the skeletal muscle, including endothelial cells, pericytes and satellite cells as well as skeletal myocytes, are potential sources of overload-induced VEGF. However, the complete lack of an increase in VEGF mRNA within the overloaded VEGF-deficient muscles indicates that myocytes constitute the key cellular source of VEGF in response to muscle overload. Abluminal sprouting of endothelial cells to generate new capillaries requires endothelial cell migration into the surrounding matrix, which is facilitated by the increased production of matrix-degrading enzymes MMP-2 and MT1-MMP [2], [4]. Consistent with *in vitro* observations in which VEGF enhances endothelial cell production of these enzymes [10], [11], we determined that muscle overload fails to enhance MMP-2 and MT1-MMP transcript levels in muscle VEGF-deficient mice. This supports the idea that VEGF acts to activate MMPs and that myocyte-derived VEGF is essential for angio-adaptation. Importantly, these data further suggest that other paracrine sources of VEGF are not capable of acting in place of, or rescuing, the loss of myocyte-derived VEGF.

The renin-angiotensin system has been implicated in adaptations following exposure of cells to mechanical forces. We found that C2C12, but not cultured endothelial cells, expressed angiotensinogen protein. *In vitro*, stretch of both proliferating and differentiated C2C12 cells elevated angiotensinogen and AT1R transcript levels [28] and *in vivo* studies examining the heart following pressure overload show elevated angiotensinogen, ACE and AT1R transcript levels [46]–[48]. Our results presented in this study reveal novel evidence that the Ang II signaling pathway may be activated in overloaded skeletal muscle, as observed by elevated angiotensinogen transcript and protein levels, and therefore may play a role in the adaptations that occur in the skeletal muscle micro-environment following overload.

Given the central role established for VEGF in overload-induced angiogenesis, we have extended the investigation on the role of myocyte-derived VEGF in modulating endothelial cell signaling using *in vitro* experiments on matrix bound VEGF secreted from C2C12 myoblasts. Here, we demonstrate that myocyte secretion of VEGF results in accumulation of matrix-associated VEGF that is capable of activating endothelial cells (Figure 3). While previous studies have activated endothelial cells using matrix–associated VEGF [18], this is the first direct evidence to our knowledge that myocyte-derived VEGF acts in this way. The deposition of matrix-associated VEGF may create a chemotactic gradient that provides guidance cues for the tip cells of nascent capillary sprouts [49], [50]. As well, MMPs can cleave the heparin sulfate binding domain contained within VEGF165 and VEGF189, releasing a soluble form of VEGF [51]. The increased production and activation of MMPs that occurs in response to muscle activity or overload [2], [4], [5], [52] would facilitate a rapid increase in diffusible VEGF within the interstitial fluid, which could further activate neighbouring satellite cells and endothelial cells.

Several lines of evidence support a role for Ang II in regulating VEGF levels, and thus, contributing to capillary homeostasis within skeletal muscle. We observed that exogenous Ang II treatment increased VEGF expression in myotubes. These cells expressed the AT1R, and we found that AT1R blockade by losartan attenuated the basal production of VEGF by myotubes. These novel results reveal the importance of Ang II signaling to basal production of VEGF in myocytes. Furthermore, exogenous Ang II stimulation of skeletal muscle endothelial cells increased cellular levels of VEGF mRNA and protein as well as MMP-2 mRNA. These findings indicate that microvascular endothelial cells are directly responsive to Ang II stimulation, confirming previous reports in resistance arteries and retinal and cardiac microvascular endothelial cells [43], [53]–[55].

Previous studies have demonstrated a role for Ang II in skeletal muscle angiogenesis (ie. in response to exercise, electrical stimulation, muscle injury and hindlimb ischemia) [23]–[25], [56], so our observation that AT1R blockade did not affect overload-induced angiogenesis was unexpected. Our data suggest that inhibition of AT1R signaling is not sufficient to reduce overall VEGF levels within the intact muscle. Animals subjected to both overload and losartan treatment had an even greater increase in VEGF expression when compared with animals treated with losartan alone, indicating that overload and losartan may act through independent pathways to raise VEGF expression in the muscle. Notably, losartan treatment alone increased VEGF mRNA in cultured endothelial cells, opposite to the response seen in myotubes, and the levels of VEGF in cells treated with Ang II and losartan were elevated when compared to VEGF levels following Ang II treatment alone. These data indicate that losartan increases endothelial cell production of VEGF, which may be a possible mechanism through which elevated levels of VEGF protein are maintained in losartan-treated rats.

While the majority of studies indicate that the AT1R mediates the Ang II-induced increases in VEGF mRNA and protein [43] and neovascularization [57], [58], a role for the AT2R in the regulation of VEGF production also has been described [59], [60]. Endothelial cells express the AT2R subtype [61] and it is feasible that losartan treatment enhanced VEGF production via enhanced signaling through the AT2R. Conflicting data on the effects of AT2R activation have been reported. On one hand, AT2R activation on endothelial cells is reported to reduce VEGF-dependent angiogenesis through inhibition of RhoA GTPase and cellular migration [61], [62]. However, several studies have provided evidence for a positive role of the AT2R in tumor angiogenesis [63] and in angiogenic sprouting in the hypoxic heart [64]. Thus, we considered it possible that Ang II signaling via the AT2R may play a role in maintaining VEGF production and the angiogenic response to muscle overload in the presence of AT1R inhibition. While we did find detectable levels of the AT2R in microvascular endothelial cells, an AT2R agonist did not influence VEGF mRNA levels. Furthermore, inhibition of the AT2R using PD123319 failed to reduce the losartan-induced increase in VEGF mRNA. Consequently, our data do not support a role for the activation of an AT2R-dependent response in the presence of losartan. The mechanism underlying the stimulatory influence of losartan treatment on the endothelial cells requires further investigation.

Alternatively, the sustained levels of VEGF in the overloaded muscle may be seen as evidence for redundancy in the pathways that lead to VEGF production in activated muscle. For example, Hoier and colleagues reported that the contraction-induced increase in VEGF mRNA and protein in humans were dependent on adenosine [22]. Also, angiogenic responses to exercise training were abolished in PGC-1α, ERRα [21] and p38γ knockout mice [65], with all animals having reduced VEGF expression. These studies and the results of our work presented here indicate that further work is required to further characterize the complexities of VEGF production in muscle in order to fully comprehend what signals regulate VEGF production downstream of the overload stimulus. Our data also emphasize the importance of specifically evaluating myocyte-derived VEGF as opposed to VEGF from other sources, such as endothelial cells. It is interesting to note, in previous work where VEGF was selectively deleted in endothelial cells, significant impairments in vascular integrity and function were found, but no measurable alterations in vascular density were noted [66]. In contrast, loss of myocyte-specific VEGF has resulted in significant losses of muscle capillarity, but without evidence of impaired vascular integrity [12], [34]. This is consistent with the current finding that overload stimulated angiogenesis is abolished in muscle VEGF deficient mice (Figure 1A, B), but more importantly would suggest that the actions of VEGF may be context and site dependent.

In summary, we have shown that Ang II signaling contributes to the basal production of VEGF by muscle cells and provides evidence that Ang II-dependent production of matrix-associated forms of VEGF is involved in the communication between muscle cells and endothelial cells. Our study also revealed that myocyte-derived VEGF is critical for new capillary growth following muscle overload. However, blockade of AT1R signaling does not reduce muscle overload-induced VEGF production and capillary growth. Together, these findings emphasize the absolute requirement for muscle-derived VEGF in the promotion of capillary growth within muscle, and indicate that the expression of this critical factor within active muscle is protected through functional redundancy of multiple signal pathways.
